# Issues in Melanoma Detection: Semisupervised Deep Learning Algorithm Development via a Combination of Human and Artificial Intelligence

**DOI:** 10.2196/39113

**Published:** 2022-12-12

**Authors:** Xinyuan Zhang, Ziqian Xie, Yang Xiang, Imran Baig, Mena Kozman, Carly Stender, Luca Giancardo, Cui Tao

**Affiliations:** 1 School of Biomedical Informatics The University of Texas Health Science Center at Houston Houston, TX United States; 2 McGovern Medical School The University of Texas Health Science Center at Houston Houston, TX United States

**Keywords:** deep learning, dermoscopic images, semisupervised learning, 3-point checklist, skin lesion, dermatology, algorithm, melanoma classification, melanoma, automatic diagnosis, skin disease

## Abstract

**Background:**

Automatic skin lesion recognition has shown to be effective in increasing access to reliable dermatology evaluation; however, most existing algorithms rely solely on images. Many diagnostic rules, including the 3-point checklist, are not considered by artificial intelligence algorithms, which comprise human knowledge and reflect the diagnosis process of human experts.

**Objective:**

In this paper, we aimed to develop a semisupervised model that can not only integrate the dermoscopic features and scoring rule from the 3-point checklist but also automate the feature-annotation process.

**Methods:**

We first trained the semisupervised model on a small, annotated data set with disease and dermoscopic feature labels and tried to improve the classification accuracy by integrating the 3-point checklist using ranking loss function. We then used a large, unlabeled data set with only disease label to learn from the trained algorithm to automatically classify skin lesions and features.

**Results:**

After adding the 3-point checklist to our model, its performance for melanoma classification improved from a mean of 0.8867 (SD 0.0191) to 0.8943 (SD 0.0115) under 5-fold cross-validation. The trained semisupervised model can automatically detect 3 dermoscopic features from the 3-point checklist, with best performances of 0.80 (area under the curve [AUC] 0.8380), 0.89 (AUC 0.9036), and 0.76 (AUC 0.8444), in some cases outperforming human annotators.

**Conclusions:**

Our proposed semisupervised learning framework can help with the automatic diagnosis of skin disease based on its ability to detect dermoscopic features and automate the label-annotation process. The framework can also help combine semantic knowledge with a computer algorithm to arrive at a more accurate and more interpretable diagnostic result, which can be applied to broader use cases.

## Introduction

Skin cancer is one of the most common cancers worldwide, with steadily increasing incidence rates of melanoma and nonmelanoma cancers [1]. Early detection of skin cancer is an important prognostic factor that can improve patient survival and overall outcomes [2]. Reliable skin cancer screening, however, may not be readily available to all patients. For example, individuals who live in rural areas without local dermatology clinics or who face barriers to attending an in-office evaluation may not have an opportunity to have skin cancer detected at an early stage. To address this concern, the use of teledermatology has become increasingly popular, particularly during the COVID-19 pandemic, which has significantly decreased in-person dermatological evaluation [3,4]. Recently, teledermatology has been shown to increase access to reliable dermatology evaluation and to minimize delays in skin cancer management [3,5]. A useful subset of teledermatology is teledermoscopy, whereby digital images of skin lesions are taken using a dermatoscopy or a smartphone with a dermatoscopy attachment [6]. Studies find that the use of dermoscopic images in teledermatology consultations improves the sensitivity and specificity of the diagnosis [3,7]. In this way, teledermoscopy offers itself as a promising tool to increase patient access to reliable skin cancer screening and, thus, the early detection of skin cancer.

The automated classification of dermoscopic images through convolutional neural networks (CNNs) has emerged as a reliable supplement to visual skin examination by on-site specialists in the detection of skin cancer [8-11]. CNNs have the potential to extend reliable skin cancer recognition to clinicians who lack special dermatology training, including nurse practitioners, physician assistants, and primary care physicians. In addition, the use of CNNs enables the evaluation of skin lesions via telemedicine. Images captured on smartphone cameras and analyzed by similar algorithms have been shown to achieve accuracy in identifying melanomas similar to that of board-certified specialists [12]. Some CNN models even exhibit greater sensitivity and specificity in diagnosing early melanoma compared with those of inexperienced clinicians [13,14].

Artificial intelligence (AI) algorithms, however, have some weaknesses. One weakness is interpretability and transparency regarding how the computer arrived at its output, making it difficult for dermatologists to trust the diagnostic results [15-17]. Another is that the current algorithms, such as the deep CNNs used in triaging and classifying suspicious skin lesions, do not provide the reasoning used to arrive at their given result [18]. This is often due to the complexity of the algorithm and hinders their utility due to a lack of the trust in the diagnosis by the patient and the physician [19].

Another limitation of AI algorithms is that a majority rely solely on images as inputs, whereas in a clinical setting, more information can be obtained through, for instance, palpation of the lesion and clinical data on age and family history [20]. The dermatologist also relies on diagnostic rules to make decisions, such as the ABCD rule, pattern analysis, 7-point checklist, and 3-point checklist, which have been developed to standardize the dermoscopic evaluation of melanoma and play a critical role in skin lesion diagnosis [8,9,21-23].

Recent studies have focused on attempts to combine semantic knowledge with the algorithm to arrive at a more accurate diagnosis [20,24-26]. Several studies have suggested that diagnoses derived using more than one source of input are more accurate than are those conceived by one method alone [27-29]. One study showed that nondermatologist physicians were able to improve their accuracy in classifying pigmented lesions when combining their knowledge of age, sex, and localization of the lesion with deep-learning frameworks [24]. Earlier research added factors such as age, body site, proportion of dysplastic nevi, naevus count, and family history of melanoma to a computer image–analysis program and found that the addition of clinical data significantly improved the ability to distinguish between benign and malignant skin lesions [30]. Another study found an improvement in the detection of basal cell carcinoma after adding factors such as lesion size and elevation, age, gender, and location [31]. Kawahara et al [32] conducted a similar work when proposing a multitask deep CNN trained on multimodal data to classify the 7-point melanoma checklist criteria and perform a skin lesion diagnosis. Even though they intergraded each feature from a 7-point checklist using loss blocks, their studies did not integrate the knowledge with the CNN architecture. One major constraint of these studies is the lack of high-quality data related to diagnosis, for example, the dermoscopic features that dermatologists use to diagnose skin lesions. In this study, we address these limitations by developing a semisupervised deep-learning framework that applies the results learned from a small, annotated data set to a larger unlabeled data set as well as by imitating the human diagnosis process in our CNN structure.

In this experiment, we chose the 3-point checklist for melanoma and melanocytic nevus as an illustration of diagnostic rules and disease class. The 3-point checklist is easy to interpret and is highly sensitive for the diagnosis of melanoma by nonexpert clinicians [33]. Melanoma is well known as the most aggressive cutaneous malignancy, accounting for approximately 75% of all skin cancer deaths [24]. It often shares morphology with melanocytic nevi on naked-eye examination, a technique that yields only 60% accuracy in a melanoma diagnosis by expert dermatologists [34]. In this regard, the International Skin Imaging Collaboration (ISIC) organizes data challenges every year, which focus primarily on diagnostic accuracy when distinguishing melanoma from other malignant and benign lesions [35]. Numerous studies that concern the use of the 3-point checklist to help classify melanomas have been conducted [33,36,37]. In these studies, participants with varying experience were able to score proven nonmelanoma and proven melanoma lesions using just the 3-point checklist criteria. A disadvantage of this method, however, is that the checklist tends to miss thinner melanomas [37]. None of the studies related to 3-point checklist has tried to combine visual inspection with CNN-extracted imaging features to arrive at a diagnosis. This is also the major difference in our state-of-the-art methodology as compared to what was seen in previous ISIC data challenges.

Combining diagnostic rules with the 3-point checklist classification algorithm can yield benefits that improve patient access to care and diagnostic accuracy. The proposed algorithms have several potential application scenarios, including the following: (1) they can automatically classify skin disease images and generate feature labels by listing the criteria used to categorize suspicious lesions to improve trust and acceptance of teledermoscopy; (2) they can assist medical students to learn and identify the features in dermoscopic images; given the detailed evaluation of each criterion in the 3-point checklist by the algorithm, students can use the checklist to learn about the fundamental parameters used to differentiate lesions as a benign nevus or a melanoma; and (3) they can automate the process of feature annotation; thus, fewer human annotators need to be involved, enabling the secondary use of enormous imaging data resources, such as the ISIC archive.

## Methods

### Data Set

All images from labeled and unlabeled data sets come from the ISIC archive. “Label” here represents the 3-point checklist feature labels, which means both “labeled” and “unlabeled” data sets contain disease type information. For the small, labeled data set, we selected an even distribution of melanoma and melanocytic nevus dermoscopic images from ISIC 2019 to annotate, using the 3-point checklist features. The large unlabeled data set came mainly from ISIC 2020, which contains the 584 melanoma and 5193 melanocytic nevus dermoscopic images. To balance the data set, we added 4062 melanoma images from ISIC 2019, excluding the images in the small, labeled data set. We divided each data set into training and validation sets in an 80/20 ratio and used 5-fold cross-validation, which means the data set was divided equally into 5 subsets and rotating in order to be the training or validation data set. We annotated an additional 400 images as a holdout testing set.

The 3-point checklist is easy to interpret and is highly sensitive for the diagnosis of melanoma versus melanocytic nevus. Our algorithm evaluated dermoscopic images of pigmented lesions based on the 3-point checklist, indicating the presence or absence of (1) asymmetry, (2) atypical pigment network, and (3) blue-white structures. If any one of these features was detected from the skin lesion image, 1 point would be added on top of the scoring for that image. The scoring range per image is 0 to 3. These 3-point automated classification outputs can aid in a provider’s decision to biopsy a lesion or to refer to a specialist for a more thorough evaluation. Table 1 presents the number of images for the skin disease categories of melanoma and melanocytic nevus.

**Table 1:** Number of images for skin disease categories for labeled and unlabeled data sets.

Disease	Unlabeled data set	Labeled data set
Melanoma	4646	450
Melanocytic nevus	5193	450
Total	9839	900

### Annotation of the 3-Point Checklist

There are 3 features of the 3-point checklist, which are atypical network, asymmetry, and blue-white structure. For each feature detected, 1 score will be added for that image. The higher the score is (usually higher than 2), the higher the risk of melanoma will be. If the score is lower than 1, according to the 3-point checklist, the lesion is more likely to be benign. Our experiment was developed based on a gold standard whereby each image was rigorously reviewed by at least 2 annotators. If consensus was reached, the resulting diagnosis was annotated. If not, a third annotator would evaluate the image again. We divided the annotation into 2 steps. First, the 3 annotators had training sessions to develop consensus annotation guidelines. We provided the annotators with a small image set annotated by domain experts to annotate and evaluate. During this phase, the annotators are allowed to discuss their different understandings. After interrater agreement reached at least 70%, we moved to the second step, in which they annotated images independently. We divided the whole image data set into 3 subsets, and each annotator was assigned 2 subsets so that every image had at least 2 annotation results. Our final interrater agreement Kappa-Cohen score for the second step was 0.64, which indicated substantial agreement. If any images had different annotation results, we brought in the third annotator, who was not previously assigned to the image, and took a majority vote. Overall, this is a very time-consuming process.

### Image Preprocessing

#### Crop and Resize

Because the training data set came from 3 data sources, each had a different resolution of the images. There could be 1 lesion that took up the entire image or just 1 corner of the graph. Hence, we developed a rule to crop and resize all the training images, which improved the performance of our model.

#### Color Constancy

Due to the different imaging sources and illuminations, the color of dermoscopic images varied considerably. Therefore, it was important to calibrate the color of the images in the preprocessing stage to reduce possible bias for the deep neural network. Catarina et al [38] compared 4 color-constancy algorithms (Gray World, max-RGB, Shades of Gray, and General Gray World) to calibrate the color of dermoscopic images for the melanoma classification system. These algorithms improved the system performance by increasing sensitivity and specificity, and Shades of Gray achieved better results than did the other color-constancy algorithms. Thus, for the project, we chose Shades of Gray as the color-constancy algorithm to calibrate the color of the dermoscopic images before the training stages. The calibration procedure involves 2 steps. First, the color of the light source in the RGB color space is estimated. Then, the image is transformed, using the estimated illuminant.

#### Contrast-Limited Adaptive Histogram Equalization

Contrast-Limited Adaptive Histogram Equalization was used to improve the contrast in images. Unlike histogram equalization, it computes several distinct sections of the image and uses them to redistribute the lightness values of the image. It helps to improve the local contrast and enhance the edges of objects in the image.

### Model Architecture

**Figure 1:**
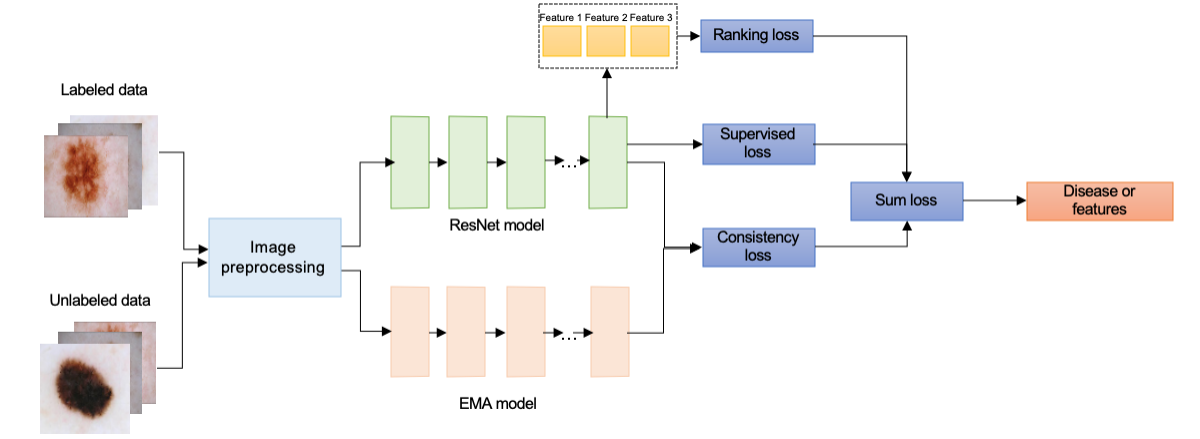
Architecture of the proposed semisupervised learning framework. EMA: exponential moving average; ResNet: residual neural network.

We proposed a semisupervised learning framework for the prediction of skin disease that uses a small set of labeled images and a larger set of unlabeled images. The labeled data set contains 900 images that were labeled with disease tags and the 3-point checklist annotation, while the unlabeled data set contains 9839 images that have only disease tags. The architecture of the proposed classification model is presented in Figure 1 and contains primarily 3 components. The input component involves the preprocessing of both labeled and unlabeled images. The output of the input component is streamed into 2 branches. One branch is the supervised learning component that uses ResNet, inside which the representation of each image is associated with the 3-point labels and the classification tag and with the label-related ranking loss [39] and classification loss, correspondingly. The other is the semisupervised learning component, whereby a consistency loss is optimized using the output from an exponential moving average (EMA) model of the ResNet branch [40]. Finally, the 3 types of losses are combined, and coefficients are used to balance their weights. We provide a detailed description of these 3 components in this section.

#### Supervised Learning + Ranking Loss

The supervised learning consists of 2 tasks, which are jointly learned during training. One task is the classification of the skin disease, and the other is the classification of each feature in the 3-point checklist. Using the 3-point checklist, each feature is given a binary score of 0 or 1 in the training phase, indicating whether it exists in the image. A total score higher than 2 suggests that the lesion is more likely to be malignant. We incorporated the traditional cross-entropy loss to optimize the skin disease classification part and used ranking loss to represent the 3-point checklist knowledge. The hyperparameters for our training models are as follows: a batch size of 128, stochastic gradient descent optimizer, and ReduceLROnPlateau learning rate decay (mode=“min,” factor=0.5, threshold=0.01, patience=7, verbose=True).

#### Semisupervised Learning

Image annotation requires not only extensive time investment but also domain expertise of human annotators. Inspired by the research of Tarvainen and Valpola [40], we developed a semisupervised scheme based on their “mean teacher” framework to automate the feature annotation process of skin lesion images. This model can use the information from small-scaled labeled images and make skin feature and disease predictions on larger unlabeled image data sets. On top of that, we developed and integrated disease- or feature-specific loss functions to combine knowledge from human expertise into the model. The predicted features can be used simultaneously in the training phase to improve the disease classification accuracy. The supervised loss is associated with the disease label of each image and denoted by the cross-entropy function. In the semisupervised learning component, the mean-teacher strategy was adopted to minimize the consistency loss between labeled and unlabeled data sets and to average the model weights from supervised and unsupervised learning.

### Theory and Calculation

#### Supervised Learning + Ranking Loss

Using the ranking loss, we enforce the model to learn a predefined diagnostic rule—the samples with higher scores are more likely to have melanoma. The ranking loss is computed from each pair of samples in a batch. We denote *o_ij_ ≡ f(x_i_)– f(x_j_)*, where *f* is the logit corresponding to the disease class, the posterior *P_ij_*, and the desired target values 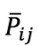:



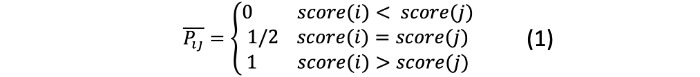



Then, the cross-entropy loss function can be represented as



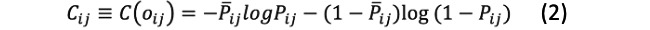



We compute *P_ij_* from *o_ij_* using the sigmoid function as follows; the loss function can be further rewritten as:



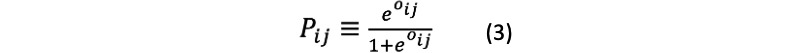





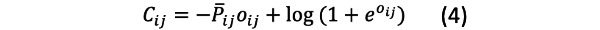



#### Semisupervised Learning

The EMA model behaves as the teacher model on the unlabeled. This method constrains the model to behave similarly to the past models during the update so it can potentially find flatter local minima and avoid singularity points where a small update would result in large behavior change in the model. The mean-teacher strategy proved useful in previous works, and the consistency cost is defined as follows, where is updated based on EMA parameters:



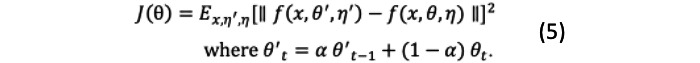



Finally, the ranking loss, disease supervised loss, feature supervised loss (FSL), and consistency loss were added together to train the model.



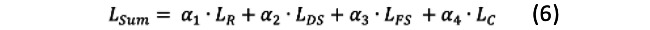



## Results

Our models were built based on the state-of-the-art ResNet model. We tried ResNet-18, ResNet-50, ResNet-152, and Resnext50_32x4d, and there was no significant difference in classification accuracies. To facilitate the training process, we used a relatively light architecture, ResNet-18, as our baseline.

The first task is to test whether the model will increase the classification accuracy after adding human knowledge, which is transformed and represented in the Ranking Loss format. Many state-of-the-art CNN model architectures have been developed for image recognition task, some of which achieved great performance on the skin lesion recognition task on ISIC data sets. In a 2021 paper published by Yiming Zhang et al [41], they reported that DenseNet [42] achieved superior performance over other deep learning approaches on the melanoma classification task using ISIC 2020 data set. MobileNet [43] is another CNN model developed in the recent years, and it has been adapted to ISIC image classification tasks in many cases [44,45]. To choose a CNN architecture as our baseline model and show the improvement of accuracy after combining the human knowledge in the ranking loss format, we compared accuracy results of the state-of-the-art CNN models mentioned above. The comparison outcomes are shown in Table 2. We chose ResNet as our baseline model for its better performance. All the models were trained using a 900 labeled data set (from Table 1). We tested the performance of pretrained baseline model on our larger 9000-image data set using 80/20 data split. The results are shown in Table 2. We used 5-fold cross-validation to calculate the mean and standard deviation of the validation accuracy.

As can be seen from the table, the pretrained baseline model reached the same level of accuracy on the large 9000-image data set. After adding the human knowledge of the 3-point checklist rule, the average accuracy even improved on this basis.

The previous experiment was based on human-annotated, 3-point feature labels. The entire process, from recruiting annotators to finally reaching agreement, took more than 2 months. Hence, we developed the semisupervised model to automate the feature-annotation process. We combined the generated features as human knowledge to test whether such knowledge can help to improve the disease classification accuracy.

To evaluate the performance of the 3-point feature classification for our semisupervised model, we calculated the testing accuracy and area under the receiving operating characteristic curve (AUC) on a separate holdout testing data set that contains 100 images with annotated 3-point features and disease type. We tested the performance for feature and disease classification on the models shown in Table 3, for which “baseline” is the labeled 900-image data set for supervised training, followed by different combinations of loss functions.

As seen in Table 3, the semisupervised model that combined all 3 loss functions achieved the best accuracy for disease classification. Adding FSL improved the performance of disease classification by 2%. The result shows that emphasizing the weight of “Asymmetry” feature improved the testing accuracy of “asymmetry” by 2% and improved the classification of the “atypical network” by 3%. Nevertheless, the accuracy of “Blue-white structure” and disease classification has a significant decrease.

**Table 2:** Five-fold cross validation results for the disease classification task.

Model	Five-fold accuracy, mean (SD)
MobileNetV3 (Pretrain=True)	0.8733 (0.0113)
DenseNet (Pretrain=True)	0.8856 (0.0114)
Baseline (ResNet-18, Pretrain=True)	0.8867 (0.0191)
Baseline + Human Knowledge (RL^a^)	0.8943 (0.0115)

^a^RL: ranking loss.

**Table 3:** Results for semisupervised model for disease or feature classification tasks with different loss functions—disease supervised loss (DSL), feature supervised loss (FSL), and consistency loss (CL).

Model	Asymmetry, accuracy (AUC^a^)	Atypical network, accuracy (AUC)	Blue-white structure, accuracy (AUC)	Disease, accuracy (AUC)
CL	0.51 (0.5760)	0.53 (0.5021)	0.54 (0.5620)	0.54 (0.5648)
DSL	0.51 (0.5480)	0.76 (0.6480)	0.58 (0.5285)	0.76 (0.8690)
FSL	0.80 (0.8380)	0.89 (0.9036)	0.74 (0.8036)	0.51 (0.5339)
FSL+CL	0.68 (0.7816)	0.87 (0.8752)	0.75 (0.8137)	0.53 (0.5402)
DSL+FSL	0.76 (0.7892)	0.86 (0.8602)	0.76 (0.8133)	0.74 (0.8418)
DSL+CL	0.53 (0.5448)	0.79 (0.4340)	0.47 (0.5943)	0.77 (0.8389)
DSL+FSL+CL	0.73 (0.8036)	0.85 (0.8474)	0.76 (0.8444)	0.79 (0.8402)
DSL+FSL^b^+CL	0.75 (0.7932)	0.88 (0.8752)	0.71 (0.7951)	0.69 (0.7971)

^a^AUC: area under the receiving operating characteristic curve.

^b^We emphasized the weight of the “Asymmetry” feature in the loss function.

## Discussion

### Annotation Process

Annotators in this study were medical students with no expert training in dermatology. They evaluated images based solely on tutorials from web-based resources and textbooks. Without any designated training, using example images, each of the annotators initially had a different idea of what each feature looked like. Preliminary agreement scores may have been improved if annotators had been given reference images from which to learn the dermoscopic features. This finding highlights the potential value of our algorithm as an educational tool. If medical students can evaluate a dermoscopic image and check their 3-point annotation against the algorithm’s validated output, it will help them develop their ability to visually identify each dermoscopic feature.

During the image-annotation process, there were some uncertainties for annotators. First, the vague definition of dermoscopic features, especially “atypical network” posed an issue, as each annotator had a different idea of what that looks like. This resulted in initial low agreement scores. We address this concern by proposing an ontology that can integrate the domain knowledge on dermoscopic features and represent the features in a more standardized, computer-readable format.

Another uncertainty in analyzing the images was the use of different screens with various color-display settings. One common error that was encountered was the inability to properly characterize blue structures when night light or blue light filters were activated. As such options can be automatically engaged on a schedule, however, this could lead to annotation errors. The use of different screens led to initial disagreement among the annotators but can be corrected by proper calibration and ensuring that no color filter is on.

One limitation of this study was that most of the images are taken from White skin. This has implications for whether the algorithm can be effective in detecting melanoma in colored skin. Training the algorithm to identify lesions in more than just one group of skin colors would be valuable in helping to screen a larger population of patients at risk of melanoma. Another limitation was that the image quality could have been decreased due to shadows, hairs, reflections, and noise, leading to an inadequate lesion analysis, as discussed in an earlier study [46].

### Classification Models

For the first task, after combining the 3-point checklist human knowledge, the loaded model weights from the large data set improved the classification accuracy from an average of 0.8867 to 0.8943. This shows that the ranking loss has a positive impact on classification accuracy. We plan to continue to work on expanding human knowledge to develop more complicated diagnostic rules to test their impacts on computer algorithms.

For the feature- and disease-classification task that used semisupervised architecture, interesting findings were discovered in Table 3. The improvement of the classification accuracy for certain feature labels can be accomplished by assigning a heavier weight on the corresponding feature’s loss function, however, at the cost of scarifying the accuracy for disease classification. Among the 3 features, blue-white structure has a relatively low accuracy when classified without feature-supervised loss function, the potential reason being the unbalance of blue-white structure data set where most of them are negative. While adding FSL is helpful for the feature classification task, adding disease-supervised loss function could bring down the performance of feature classification. For the disease classification, adding FSL alone did not improve the accuracy; however, combining consistency loss with FSL is showing a positive effect on disease classification.

We also noticed that, during the human annotation process for the 3-point checklist, the atypical network had the lowest interagreement rate among the 3 annotators. For the computer feature-classification task, however, the atypical network had the highest classification accuracy. This suggests that the algorithm has the advantage of learning certain image features that might be a challenge for human experts. This shows that human intelligence and AI can complement each other.

Because our image data set is from the ISIC archives, we also compared the performance of our algorithm with the winner of the ISIC 2020 leaderboard [47]. The current best performance has an AUC of 0.949. The AUC of the proposed model on the 400 unlabeled-image testing set (from ISIC 2020) is 0.9848 with different settings of disease category. Our 0.9848 AUC, however, cannot be directly compared with the results from the ISIC leaderboard, as our classification task includes only melanoma and melanocytic nevus, whereas the ISIC challenge has some “unknown” images. The remainder of the results in this regard are calculated on the small 100 labeled-image testing set, which has significant improvement over the application of the student-teacher framework, indicating the power of semisupervised learning.

### Future Steps

We plan to implement more fine-tuned model architectures trained from scratch so that a more advanced ensemble can be applied by integrating architectures from submodels. Our current experimental setting for the disease classes and rules of the 3-point checklist is only a demonstration of how we can integrate the human thinking process into the structure of CNNs. There are numerous diagnostic rules that are being developed, as dermatology is thriving, and we plan to summarize all the diagnostic rules and dermoscopic features mentioned, as well as their relationship with skin diseases, into ontology and to further accelerate the automation process of clinical decision support by computer algorithms. With our trained algorithm, we can already automate the 3-point checklist annotation process and apply it to a wider range of image databases.

### Conclusions

This study is distinctive because it combines the semantic knowledge from the 3-point checklist with a computer algorithm (CNN) to arrive at a more accurate and more interpretable diagnosis. The CNN classification was conducted based on more information than just the imaging pixels. Due to the time and labor consumption of the image-annotation process, there are vast imaging data sets that remain undiscovered. Our proposed semisupervised learning framework can help automate the annotation process, enabling the reuse of many skin-imaging data sets, which is also beneficial to the robustness and domain adaptation of the deep-learning model.

## Abbreviations

AI: artificial intelligence
AUC: area under the receiving operating characteristic curve
CNN: convolutional neural network
EMA: exponential moving average
FSL: feature supervised loss
ISIC: International Skin Imaging Collaboration
